# MBOAT7 rs641738 variant and hepatocellular carcinoma in non-cirrhotic individuals

**DOI:** 10.1038/s41598-017-04991-0

**Published:** 2017-07-03

**Authors:** Benedetta Donati, Paola Dongiovanni, Stefano Romeo, Marica Meroni, Misti McCain, Luca Miele, Salvatore Petta, Silvia Maier, Chiara Rosso, Laura De Luca, Ester Vanni, Stefania Grimaudo, Renato Romagnoli, Fabio Colli, Flaminia Ferri, Rosellina Margherita Mancina, Paula Iruzubieta, Antonio Craxi, Anna Ludovica Fracanzani, Antonio Grieco, Stefano Ginanni Corradini, Alessio Aghemo, Massimo Colombo, Giorgio Soardo, Elisabetta Bugianesi, Helen Reeves, Quentin M. Anstee, Silvia Fargion, Luca Valenti

**Affiliations:** 1 Department of Pathophysiology and Transplantation, Università degli Studi di Milano, 20122 Milano, Italy; 2 Internal Medicine, Fondazione IRCCS Ca’ Granda Ospedale Policlinico Milano, Milano, Italy; 3 Sahlgrenska Center for Cardiovascular and Metabolic Research, Wallenberg Laboratory, Department of Molecular and Clinical Medicine, Department of Cardiology, University of Gothenburg, Gothenburg, Sweden; 4 Clinical Nutrition Unit, Department of Medical and Surgical Sciences, University Magna Graecia, Catanzaro, Italy; 5 Northern Institute of Cancer Research, The Medical School, Newcastle University, Newcastle-upon-Tyne, UK; 6 Hepatology, Newcastle upon Tyne Hospitals NHS Foundation Trust, Newcastle upon Tyne, UK; 7 Internal Medicine, Policlinico Gemelli, Roma, Italy; 8 Department of Gastroenterology, Università di Palermo, Palermo, Italy; 9 Internal Medicine and Liver Unit, Department of Experimental and Clinical Medical Sciences, University of Udine, Udine, Italy; 10 Gastroenterology, Department of Medical Sciences, University of Turin, Turin, Italy; 11 General Surgery and Liver Transplantation Center, Department of Surgical Sciences, University of Turin, Turin, Italy; 12 Department of Clinical Medicine, Gastroenterology division, Sapienza University, Rome, Italy; 13 Liver Research Group, Institute of Cellular Medicine, The Medical School, Newcastle University, Newcastle-upon-Tyne, UK; 14 Gastroenterology and Hepatology, Fondazione IRCCS Ca’ Granda Ospedale Policlinico Milano, Milano, Italy; 15 Hepatology, Humanitas Clinical and Research Center, Rozzano, Italy

## Abstract

Nonalcoholic fatty liver disease (NAFLD) represents an emerging cause of hepatocellular carcinoma (HCC), especially in non-cirrhotic individuals. The rs641738 C > T *MBOAT7/TMC4* variant predisposes to progressive NAFLD, but the impact on hepatic carcinogenesis is unknown. In Italian NAFLD patients, the rs641738 T allele was associated with NAFLD-HCC (OR 1.65, 1.08–2.55; n = 765), particularly in those without advanced fibrosis (p < 0.001). The risk T allele was linked to 3’-UTR variation in MBOAT7 and to reduced MBOAT7 expression in patients without severe fibrosis. The number of *PNPLA3*, *TM6SF2*, and *MBOAT7* risk variants was associated with NAFLD-HCC independently of clinical factors (p < 0.001), but did not significantly improve their predictive accuracy. When combining data from an independent UK NAFLD cohort, in the overall cohort of non-cirrhotic patients (n = 913, 41 with HCC) the T allele remained associated with HCC (OR 2.10, 1.33–3.31). Finally, in a combined cohort of non-cirrhotic patients with chronic hepatitis C or alcoholic liver disease (n = 1121), the T allele was independently associated with HCC risk (OR 1.93, 1.07–3.58). In conclusion, the *MBOAT7* rs641738 T allele is associated with reduced MBOAT7 expression and may predispose to HCC in patients without cirrhosis, suggesting it should be evaluated in future prospective studies aimed at stratifying NAFLD-HCC risk.

## Introduction

Following the epidemics of obesity and insulin resistance and the recent improvements in the prevention and treatment of viral hepatitis, nonalcoholic fatty liver disease (NAFLD) is becoming a major cause of hepatocellular carcinoma (HCC) in Western countries^1–4^. NAFLD affects 16–38% of the general population worldwide^5^ and is leading cause of cirrhosis, the main risk factor for HCC. However, HCC frequently develops in NAFLD patients even in the absence of severe liver fibrosis^6^. The high prevalence of NAFLD and development of HCC in non-cirrhotic patients unaware of being at risk renders therefore classic HCC screening strategies aimed at early diagnosis and curative treatment^7^ unfeasible. Thus, there is an urgent need of non-invasive biomarkers able to stratify NAFLD-HCC risk, especially in patients without severe fibrosis^8, 9^.

Inherited factors contribute to HCC susceptibility, and strong familial aggregation is observed^10^. NAFLD too has a strong heritable component^11^, and the *PNPLA3* I148M variant is the main common genetic determinant of hepatic fat content and of progressive NAFLD^12–15^. The mechanism is related to accumulation of the mutated protein^16^, which interferes with lipid droplets remodeling in hepatocytes^15, 17, 18^, and with retinol release by hepatic stellate cells^19, 20^. The *PNPLA3* variant predicts HCC development in European patients with NAFLD^21^, suggesting that genetic risk factors may prove helpful to select high-risk individuals for screening^21–23^, but has a low specificity to be used as single prognostic biomarker^24^. Furthermore, the *PNPLA3* variant also predisposes to HCC in other liver diseases associated with steatosis, namely alcoholic liver disease (ALD) and chronic hepatitis C (CHC)^23^. The *TM6SF2* E167K variant also predisposes to progressive NAFLD by altering the secretion of very low-density lipoproteins^25–27^, but its direct role in HCC predisposition is disputed^26, 28^.

The *MBOAT7/TMC4* locus rs641738 C > T sequence variant predisposes to liver fibrosis development in individuals with excessive alcohol intake^29^ and chronic hepatitis C^30^, and to the development and the progression of NAFLD in individuals of European descent^31, 32^. However, whether the rs641738 variant is also associated with HCC risk is still unknown. Aim of this study was therefore to evaluate whether the rs641738 variant predisposes to HCC in NAFLD patients stratified by the presence of severe fibrosis, and in other liver diseases characterized by hepatic steatosis.

## Results

### The Italian NAFLD cohort

The clinical features of Italian NAFLD patients stratified by HCC diagnosis are presented in Table 1. Patients who developed HCC were older (p < 0.001), had higher prevalence of type 2 diabetes (T2DM; p < 0.001), and of severe fibrosis (stage F3-F4) than those who did not (Table 1), whereas sex distribution and prevalence of obesity were not different (p = NS). Concerning genetic risk factors, at univariate analysis HCC development was associated with *PNPLA3* and *MBOAT7* variants (p < 0.001 and p = 0.003, respectively; Table 1).

**Table 1:** Clinical features of 765 Italian NAFLD patients stratified by HCC diagnosis.

	Hepatocellular carcinoma		p
	Yes (n = 132)	No (n = 633)	
Age, years	67.0 ± 8.8	47.5 ± 12.3	<0.001
Sex, Female	25 (19)	163 (26)	0.25
Obesity, yes	39 (32)	202 (32)	0.99
T2DM, yes	84 (64)	124 (20)	<0.001
Severe fibrosis, F3-4	111 (84)	99 (16)	<0.001
*PNPLA3*, I148M			<0.001
I/I	31 (23)	231 (36)	
I/M	55 (42)	283 (45)	
M/M	46 (35)	120 (19)	
*TM6SF2*, E167K			0.30
E/E	109 (83)	538 (85)	
E/K	19 (14)	88 (14)	
K/K	4 (3)	7 (1)	
*MBOAT7*, rs641738 C > T			0.003
C/C	26 (20)	213 (34)	
C/T	69 (52)	285 (45)	
T/T	37 (28)	135 (21)	

(): % values; T2DM: type 2 diabetes mellitus. Comparisons were performed by logistic regression setting HCC as dependent variable, and the association of genetic variants was analyzed assuming additive models.

The clinical features of Italian NAFLD-HCC patients according to presence of severe fibrosis are reported in Table S1. Patients who developed HCC in the absence of severe fibrosis (n = 21, 17%) were more frequently males (p = 0.040), and carriers of the *MBOAT7* rs641738 risk T allele (p = 0.006).

### *MBOAT7* variation is associated with NAFLD-HCC in Italian patients

**Figure 1:**
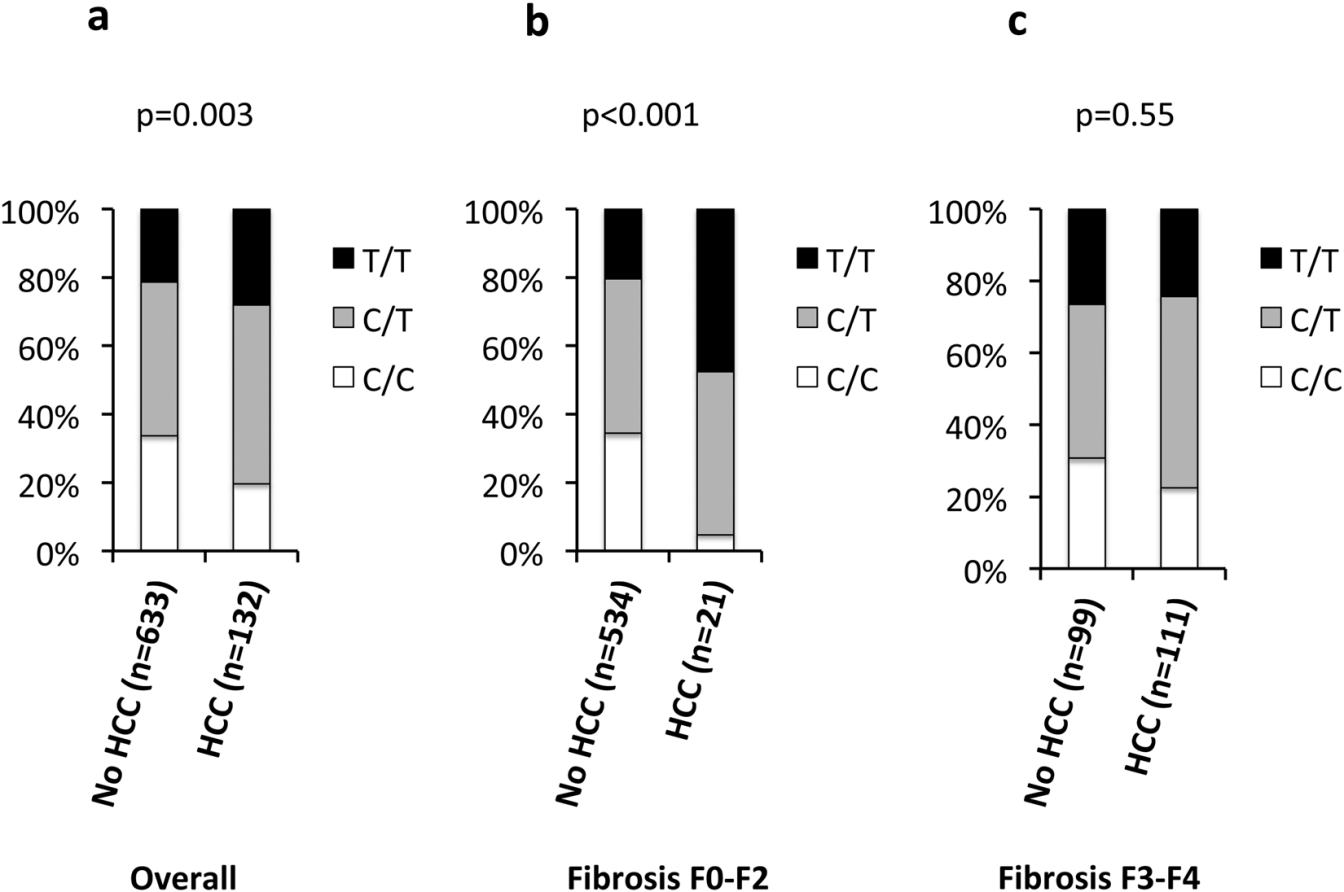
Frequency distribution of the *MBOAT7* locus rs641738 T allele in 765 Italian NAFLD patients stratified by the presence of hepatocellular carcinoma (HCC). (**a**) Overall cohort; (**b**) patients with stage F0-F2 fibrosis; (**c**) patients with stage F3-F4 fibrosis. Comparisons were performed by logistic regression setting HCC as dependent variable, and the association with the *MBOAT7* variant was analyzed assuming an additive model.

The frequency distribution of the *MBOAT7* rs641738 C > T polymorphism in Italian NAFLD patients stratified by the presence of HCC is shown in Fig. 1A. There was a significant over-representation of the rs641738 T allele in HCC vs. non-HCC NAFLD patients (p = 0.003, Fig. 1a and Table 1). In the NAFLD population, which was selected due to referral for suspected steatohepatitis/HCC, there was a borderline deviation from Hardy-Weinberg equilibrium for the frequency distribution of the rs641738 *MBOAT7* variant (p = 0.03 in both groups). However, the frequency distribution of the rs641738 variant did not violate Hardy-Weinberg equilibrium in 243 unselected healthy control subjects (p = NS; Table S3).

The clinical features of Italian NAFLD patients stratified by the rs641738 genotype are shown in Table S2. The rs641738 T allele was borderline associated with T2DM (p = 0.05) in the overall cohort, but this was not confirmed in patients stratified by HCC diagnosis, and was possibly explained by the confounding effect of the association between both rs641738 T allele and T2DM with HCC. In HCC patients, the T allele was associated with obesity (p = 0.035), and lack of severe fibrosis F3-F4 (p = 0.006).

As expected, the rs641738 T allele was nearly associated with severe fibrosis (stage F3-F4; OR 1.24, 95% c.i. 1.00–1.54; p = 0.052). The frequency distribution of *PNPLA3*, *TM6SF2*, and *MBOAT7* variants according to hepatocellular carcinoma (HCC) diagnosis in Italian NAFLD patients stratified by the severity of fibrosis (stage F0-F2 vs. F3-F4) is presented in Table 2. While the *PNPLA3* variant was associated with HCC development in patients with (p = 0.011), but not in those without severe fibrosis (p = NS), the *MBOAT7* T allele was associated with HCC in patients without (p < 0.001; Fig. 1b and Table 2), but not in those with (p = 0.55; Fig. 1c and Table 2) severe fibrosis.

**Table 2:** Frequency distribution of *PNPLA3*, *TM6SF2*, and *MBOAT7* variants according to hepatocellular carcinoma (HCC) in 765 Italian NAFLD patients stratified by the severity of fibrosis (stage F0-F2 vs. F3-F4).

Fibrosis stage						
HCC	F0-F2		p	F3-F4		p
	Yes	No		Yes	No	
	Yes (n = 21)	No (n = 534)		Yes (n = 111)	No (n = 99)	
PNPLA3, I148M			0.92			0.011
I/I	10 (48)	200 (37)		21 (19)	31 (31)	
I/M	6 (28)	236 (44)		49 (44)	47 (48)	
M/M	5 (24)	98 (19)		41 (37)	22 (22)	
TM6SF2, E167K			0.11			0.50
E/E	16 (76)	458 (86)		93 (84)	80 (80)	
E/K	4 (19)	70 (13)		15 (13)	18 (19)	
K/K	1 (5)	6 (1)		3 (3)	1 (1)	
*MBOAT7*, rs641738			<0.001			0.55
C/C	1 (4)	183 (35)		25 (22)	30 (30)	
C/T	10 (48)	243 (45)		59 (53)	42 (43)	
T/T	10 (48)	108 (20)		27 (25)	27 (27)	

(): % values. HCC. Hepatocellular carcinoma. Comparisons were performed by logistic regression setting HCC as dependent variable, and the association of genetic variants was analyzed assuming additive models.

### Independent predictors of NAFLD-HCC

The independent predictors of NAFLD-HCC are presented in Table 3. At univariate analysis (left panel), development of HCC was associated with older age, T2DM, and severe fibrosis (p < 0.001 for all), whereas among the genetic factors with *PNPLA3* I148M (p < 0.001) and *MBOAT7* T rs641738 T alleles (OR 2.18, 95% c.i. 1.30–3.63; p = 0.003).

**Table 3:** Independent predictors of HCC in 765 Italian patients with NAFLD.

	Unadjusted			Model 1			Model 2		
	OR	95% c.i.	p	OR	95% c.i.	p	OR	95% c.i.	p
Age, years	1.20	1.16–1.24	<0.001	1.19	1.15–1.23	<0.001	1.16	1.11–1.21	<0.001
Sex, Female	0.57	0.41–1.06	0.09	0.52	0.27–0.99	0.045	0.45	0.21–0.91	0.026
Obesity, yes	1.11	0.75–1.69	0.61	1.69	1.05–2.56	0.069	2.50	1.32–4.76	0.008
T2DM, yes	7.18	4.81–10.84	<0.001	4.73	2.75–8.30	<0.001	3.33	1.75–6.44	<0.001
Severe fibrosis, F3-F4	28.9	17.6–49.5	<0.001	NA	NA	NA	12.5	6.36–6.1	<0.001
*PNPLA3*, n I148M alleles	1.70	1.32–2.21	<0.001	1.61	1.12–2.32	0.010	1.31	0.86–2.03	0.24
*TM6SF2*, n E167K alleles	1.27	0.82–1.92	0.27	1.99	1.08–3.65	0.027	2.80	1.33–6.10	0.008
*MBOAT7*, n T alleles	2.18	1.30–3.63	0.003	1.81	1.24–2.69	0.002	1.65	1.08–2.55	0.021

OR: odds ratio of HCC, 95% c.i.: 95% confidence interval; T2DM: type 2 diabetes mellitus; n: number of at risk alleles. Comparisons were performed by logistic regression setting HCC as dependent variable, and the association of genetic variants was analyzed assuming additive models. Model 1: adjusted for age, sex, obesity, and T2DM; Model 2: further adjusted for presence of advanced fibrosis; NA: not addressed.

At multivariate logistic regression analysis including as independent variables noninvasive predictors of HCC (Model 1, middle panel), which can be applied even in NAFLD patients without histological evaluation of liver damage, HCC development was associated with older age (p < 0.001), male sex (p = 0.045), T2DM (p < 0.001), *PNPLA3* I148M alleles (p = 0.010), *TM6SF2* E167K alleles (p = 0.027), and remained strongly associated with *MBOAT7* rs641738 alleles (OR 1.81, 95% c.i. 1.24–2.69; p = 0.002).

After further adjustment for the presence of severe fibrosis stage F3-F4 (Model 2), the *TM6SF2* E167K (p = 0.008) and *MBOAT7* rs641738 T (OR per allele 1.65, 95% c.i. 1.08–2.55; p = 0.021; OR for T/T vs. C/C 2.73, 95% c.i. 1.17–6.51, p = 0.008) alleles remained significantly associated with HCC risk, whereas the effect of the *PNPLA3* I148M variant was lost.

In Model 1, the effect of MBOAT7 variant was larger in patients without severe fibrosis (OR per allele 2.78, 95% c.i. 1.04–8.71; p = 0.050), whereas it was not significant considering only patients with severe fibrosis (OR per allele 1.19, 95% c.i. 0.78–2.03; p = 0.3).

### Combined effect of genetic risk factors for NAFLD-HCC

**Figure 2:**
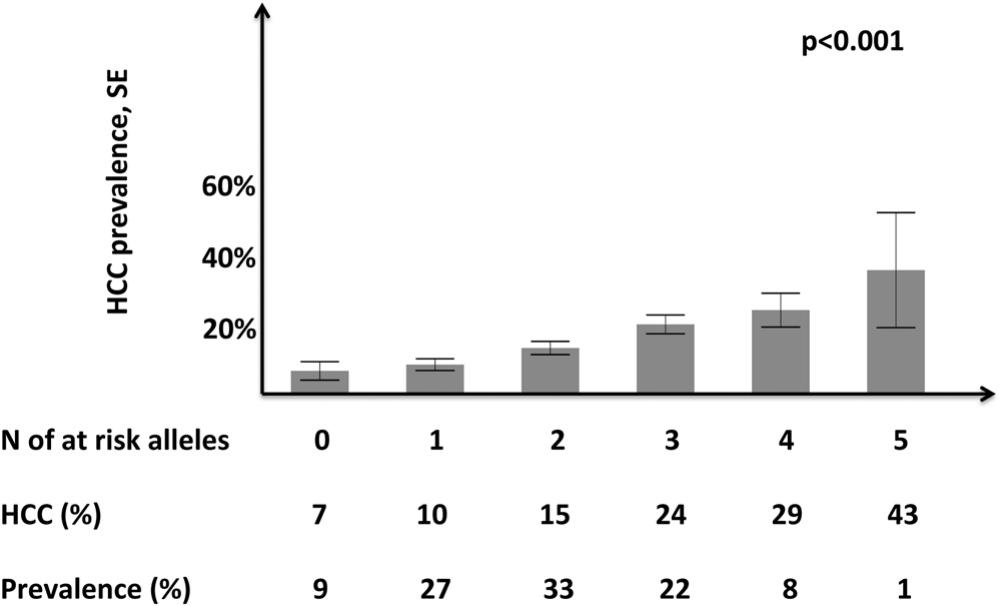
Risk of hepatocellular carcinoma according to the number of *PNPLA3* I148M, *TM6SF2* E167K, and *MBOAT7* rs641738 C > T risk variants in 765 Italian patients with NAFLD. HCC: hepatocellular carcinoma; SE: standard error. Comparisons were performed by a multivariate logistic regression setting HCC as dependent variable, and the association with genetic risk factors (numbers of at risk alleles carried) was analyzed assuming an additive model. p < 0.001 for the association of the number of risk alleles with HCC, both at unadjusted analysis and after adjustment for age, sex, obesity, T2DM, and presence of advanced fibrosis stage F2-F4.

The relationship between the total number of risk alleles including *PNPLA3* I148M, *TM6SF2* E167K, and *MBOAT7* rs641738 T and HCC risk is presented in Fig. 2. There was a significant association between the number of risk alleles and HCC (OR per allele 1.56, 95% c.i. 1.31–1.86; OR 9.25, 95% c.i. 3.83–22.8 between the extremes of the distribution, i.e. 5 vs. 0 risk alleles; p < 0.001 for both). HCC risk was 9% in the 36% of the population with 0–1 risk alleles, 19% in the 55% of the population with 2–3 risk alleles, and 31% in the 9% of the population with 4–5 risk alleles. The association held constant after correction for other risk factors as in Model 2 (OR per allele 1.68, 95% c.i. 1.30–2.20; OR 13.4, 95% c.i. 3.71–51.5 between the extremes of the distribution; p < 0.001 for both).

A combined risk score considering acquired and genetic risk factors was developed to predict HCC: 1/(1 + e^−^ ((−12.588 + (0.162 * age) + (0.404 * Sex: 1 if male, −1 if female) + (0.259 * Obesity: 1 present, −1 absent) + (0.587 * T2DM: 1 present, −1 absent) + (1.299 * Severe Fibrosis: 1 yes, −1 no) + (0.442 * number of risk alleles))). The model had a 0.96 ± 0.4 area under the receiving operating characteristic curve (AUROC) for detecting HCC cases. The optimal cutoff (identifying the best combination of sensitivity and specificity) had 96% sensitivity and 89% specificity for HCC (Fig. S1). The corresponding AUROC of a model taking into consideration only clinical factors was slightly lower (0.93 ± 0.5). In the subgroup of patients without severe fibrosis, the AUROC for clinical factors alone was 0.91 ± 0.5, whereas the full model incorporating genetic risk factors maintained an AUROC of 0.96 ± 0.4 (p = NS vs. clinical factors alone).

### Relationship between *MBOAT7* locus variants and gene expression

To investigate the biological basis for the stronger association of MBOAT7 locus variation with HCC in patients without severe fibrosis, we next examined the association of the rs641738 variant with possibly variants that may influence MBOAT7 mRNA stability, and with hepatic MBOAT7 mRNA expression levels in patients stratified by the severity of liver fibrosis.

In 98 severely obese patients, the rs641738 variant was in high linkage with the MBOAT7 3′-UTR variant rs8736 C > T polymorphism (R^2^ = 0.98; only 1/98 discordant case). Despite this, the rs8736 polymorphism was non-significantly more closely associated with NAFLD (p = 0.048 vs. p = 0.057) and MBOAT7 expression (p = 0.042 vs. p = 0.046) than rs641738. These data are in line with the hypothesis that rs641738 is not the causal variant, but may be in linkage with variants influencing MBOAT7 expression.

**Figure 3:**
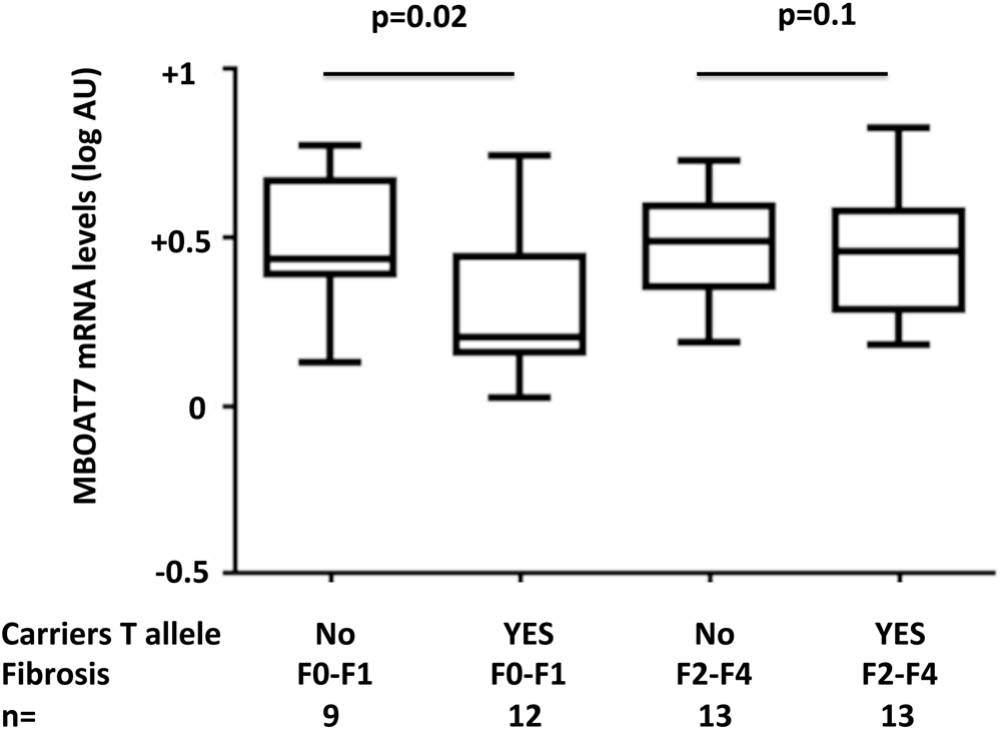
Impact of the presence of rs643718 risk T allele on MBOAT7 expression (log mRNA levels) in 47 patients with NAFLD from the Milan Hepatology service stratified by the presence of clinically significant hepatic fibrosis (stage F2-F4). Data were compared by Student’s t-test.

Gene expression of MBOAT7 in 47 patients from the Hepatology service characterized by more severe liver damage (Table S4) is shown in Fig. 3. The rs641738 T allele was associated with reduced hepatic MBOAT7 expression in patients absent or mild fibrosis (stage F0-F1; p = 0.02), but not in those with moderate-severe fibrosis (stage F2-F4; p = 0.1).

### *MBOAT7* variation and NAFLD-HCC risk in UK non-cirrhotic NAFLD patients

To increase the study power, we next evaluated the association of the rs641738 T allele with HCC risk in non-cirrhotic NAFLD patients from UK, whose clinical features are presented in Table S5. The frequency distribution of the rs641738 C > T genotype in patients stratified by HCC diagnosis is shown in Table 4. Although in the UK non-cirrhotic NAFLD cohort (N = 358, of whom 20 with HCC) *MBOAT7* variation was not significantly associated with HCC (p = 0.32), in the overall combined UK/Italian cohort of NAFLD patients without advanced fibrosis/cirrhosis the T allele remained associated with an increased risk of HCC (allelic OR 2.10, 95% c.i. 1.33–3.31).

**Table 4:** Frequency distribution of the rs641738 C > T genotype in 913 patients without advanced liver fibrosis/cirrhosis from the Italian and UK cohorts stratified by HCC diagnosis.

Hepatocellular carcinoma				
	YES	NO	p*	OR (95% c.i.)
Italy, n=	21	534		
*MBOAT7*, rs641738 C > T			<0.001	3.09 (1.58–6.02)
C/C	1 (4)	183 (35)		
C/T	10 (48)	243 (45)		
T/T	10 (48)	108 (20)		
UK, n=	20	338		
*MBOAT7*, rs641738 C > T			0.32	1.39 (0.71–2.71)
C/C	4 (20)	97 (29)		
C/T	11 (55)	179 (53)		
T/T	5 (25)	62 (18)		
OVERALL, n=	41	872		
MBOAT7, rs641738 C > T			0.001	2.10 (1.33–3.31)
C/C	5 (12)	280 (32)		
C/T	21 (51)	422 (49)		
T/T	15 (37)	170 (19)		

*Comparisons were performed by logistic regression setting HCC as dependent variable, and the association of genetic variants was analyzed assuming additive models. (): % values; OR, 95% c.i.: allelic odds ratio and 95% confidence intervals.

### Impact of *MBOAT7* variation on HCC risk in non-cirrhotic patients with other liver diseases

We finally evaluated the impact of the rs641738 T allele on HCC risk in 1121 non-cirrhotic patients with CHC and ALD (25, 2% with HCC, Table S6). Results are presented in Table 5. The rs641738 T allele was associated with increased risk of HCC, independently of age, sex, and the etiology of liver disease (OR 1.93 for each T allele, 95% c.i. 1.07–3.58; p = 0.035), with an effect size comparable to that observed in NAFLD. We observed a similar trend for association of the T allele with non-cirrhotic HCC in patients with CHC and ALD analyzed separately (Table S7). The *PNPLA3* I148M variant was also associated with HCC development outside cirrhosis, with a similar effect size of that of MBOAT7 variation (Table 5; p = 0.021).

**Table 5:** Independent predictors of hepatocellular carcinoma (HCC) in 1121 non-cirrhotic patients with chronic liver diseases associated with hepatic fat accumulation (597 with chronic hepatitis C and 524 with alcoholic liver disease).

HCC					
	No (n = 1096)	Yes (n = 25)	p value	Allelic OR, 95% c.i.*	p value*
*PNPLA3* I148M	558/437/101 (51/40/9)	8/10/7 (32/40/28)	0.016	1.92, 1.07–3.45	0.021
*TM6SF2* E167K	973/122/1 (89/11/0)	20/5 (80/20)	0.16	1.96, 0.61–5.27	0.16
*MBOAT7/TMC4* rs641738 C > T	327/510/259 (30/46/24)	2/15/8 (8/60/32)	0.028	1.93, 1.07–3.58	0.035

(): % values; OR: odds ratio; c.i.: confidence interval. Comparisons were performed by logistic regression setting HCC as dependent variable, and the association of genetic variants was analyzed assuming additive models. * Adjusted for age, sex, liver disease etiology, and *PNPLA3*, *TM6SF2* and *MBOAT7/TMC4* genetic variants.

## Discussion

In this study, we evaluated whether the rs641738 C > T *MBOAT7* locus sequence variant, associated with the development and progression of NAFLD^31, 32^, influences susceptibility to NAFLD-HCC. The main result is that in the Italian NAFLD cohort each *MBOAT7* rs641738 T allele conferred an approximately 80% increased risk of HCC. The association was mostly driven by a strong enrichment in the risk T allele in patients without advanced liver fibrosis, suggesting that *MBOAT7* variation predisposes to HCC development particularly in non-cirrhotic patients. To confirm this hypothesis, we also examined the frequency of the *MBOAT7* T allele in an independent UK cohort of non-cirrhotic NAFLD patients. Although the T allele was not significantly associated with NAFLD-HCC in this replication cohort, it remained significant in the combined cohort of 913 non-cirrhotic European NAFLD patients (41 with HCC) where it was associated with a greater than 2-fold increased risk of NAFLD-HCC; a relatively large effect size for a common genetic variant. This genetic polymorphism might thus represent a first useful biomarker to stratify HCC risk among individuals affected by NAFLD without advanced liver fibrosis.

The MBOAT7 protein catalyzes the transfer of polyunsaturated fatty acids such as arachidonoyl-CoA to lyso-phosphatidylinositol, thereby allowing to achieve an adequate level of desaturation in cell membranes^31^. The rs641738 T allele is associated with reduced MBOAT7 expression and altered phosphatidyl-inositol plasma and hepatic composition^31, 32^, favoring hepatocellular fat accumulation and the production of inflammatory mediators^31^. However, rs641738 is not likely the causal variant underpinning susceptibility to NAFLD and HCC, as we observed that it is in strong linkage with other polymorphisms in 3′-UTR of MBOAT7, which may be more closely related to the phenotype and are potentially involved in the regulation of MBOAT7 mRNA stability.

It could be speculated that the effect size of rs641738 on HCC risk was larger in patients without severe fibrosis because the presence of the risk variant may somewhat compensate for the lack of the cirrhotic pro-carcinogenic environment. However, we observed that the rs641738 T allele is associated with reduced hepatic expression of MBOAT7^31^ only in NAFLD patients without severe fibrosis. Therefore, the *MBOAT7* variant may exert its deleterious effect specifically at early stages of liver disease. Alteration of hepatic parenchymal structure and relative cell-types representation may then hamper the impact of the *MBOAT7* variant during severe fibrosis, because MBOAT7 is highly expressed in hepatic stellate cells and inflammatory cells^31, 33^. In keeping with this interpretation, the rs641738 T allele was also associated with development of early stages, but not severe fibrosis in patients at risk of NASH^31^, and in a large cohort of CHC patients^30^. Therefore, the *MBOAT7* variation might have a dual impact on liver disease during initial stages: either predisposes to HCC development before severe fibrosis ensues, or it facilitates the evolution to early-intermediate fibrosis. In line with this hypothesis, we also showed that the rs641738 T allele was associated with HCC development in non-cirrhotic patients with ALD or CHC.

In the Italian NAFLD cohort, the overall impact of the *MBOAT7* rs641738 on HCC risk was similar to that of the I148M *PNPLA3* variant. However, the effect of the I148M variant on HCC risk was not independent of severe fibrosis, suggesting that the mechanism is partly mediated by promotion of hepatic fibrogenesis and alteration of hepatic stellate cells biology^15, 19, 34^. Notably, the size effect of the *PNPLA3* I148M variant was larger and only partially attenuated by the impact on liver fibrosis in a previous study conducted in a UK cohort^21^. This difference may be due to lifestyle factors, and to the higher prevalence of clinical cofactors, as opposed to genetic risk variants (lower frequency of the I148M variant) in the UK cohort. In addition, we also report for the first time an association between the *TM6SF2* E167K variant and NAFLD-HCC. However, this association was not detected by univariate analysis due to an interaction of the *TM6SF2* variant with clinical factors, and it has previously been absent when sought in the UK NAFLD cohort^26^, whereas a predisposing effect on HCC was reported in a Italian cohort of patients with alcoholic cirrhosis^28^. Further studies are therefore required to confirm whether the E167K variant is an independent risk factor for HCC.

All in all, data suggest that genetic variants predisposing to hepatic fat accumulation promote hepatic carcinogenesis. Indeed, hepatocellular fat accumulation represents a key feature of hepatic carcinogenesis^35, 36^. Therefore, they might represent useful biomarkers for risk stratification. In fact, in the Italian NAFLD cohort the number of genetic risk variants carried was strongly associated with HCC, with 13.4-fold higher risk in those carrying five risk variants as compared to none. Remarkably, the number of genetic risk variants was able to classify NAFLD patients in three groups with different HCC risk: 9% in the 36% of patients with 0–1 risk alleles, l9% in the 55% with 2–3 risk alleles, and 31% in those carrying more than 3 risk alleles. This could in principle allow a better stratification of HCC risk than those allowed by the *PNPLA3* I148M variant alone, which was proposed by the EASL-EASD-EASO NAFLD guidelines^21, 37^. However, in the present cross-sectional Italian cohort genetic risk variants did not significantly improve the predictive accuracy of clinical factors.

Limitations of the study include its cross-sectional retrospective nature, resulting similarly to previous reports^21^ in an uneven representation of clinical risk factors (age, sex, T2DM, severe fibrosis) between HCC cases and controls. However, the majority of NAFLD-HCC patients are still diagnosed incidentally outside regular follow-up^38^, so that prospective studies in patients with advanced disease would not be more informative, especially for the risk of non-cirrhotic NAFLD-HCC. This could have led to an underestimation of the impact of inherited genetic risk variants on NAFLD-HCC, whereas the impact of clinical risk factors may have been overestimated. Therefore, the weight of specific factors in determining the HCC risk score should be reassessed in larger prospective cohorts with long follow-up and availability of the genetic risk profile before evaluation of genetic risk variants can be considered for implementation in clinical practice. Due to the relatively low number of patients included, these limitations are particularly relevant for the association of MBOAT7 variation with HCC development in patients without severe liver fibrosis. Finally, these results may not be applicable to other ethnic groups.

In conclusion, the *MBOAT7* rs641738 T allele is associated with reduced MBOAT7 expression and may predispose to HCC in European individuals without cirrhosis, suggesting it should be evaluated in future prospective studies aimed at stratifying NAFLD-HCC risk.

## Methods

### Patients

We enrolled 132 consecutive unrelated patients with NAFLD-HCC of Italian descent, referred between January 2008 and January 2015 to the Milan, Udine, Turin, Rome, and Palermo hospitals, for whom DNA samples were available. Diagnosis of HCC was based on the EASL–EORTC Clinical Practice Guidelines^7^. In the absence of liver biopsy, diagnosis of NAFLD required detection of ultrasonographic steatosis plus at least one criterion of the metabolic syndrome.

As controls, we selected Italian-ancestry patients with histologically confirmed NAFLD followed at the same referral outpatient Hepatology services during the same study period^27, 39^, from a recently published database^31^, who did not develop HCC during follow-up. We did not consider for this study bariatric patients from Milan because of the different recruitment criteria (indication to metabolic surgery), leading to different demographic and clinical features^27, 39^, and the lack of incident HCC cases in the cohort.

All patients were tested for secondary causes of steatosis including alcohol abuse (≥30/20 g/day in M/F) and the use of drugs known to precipitate steatosis. Viral and autoimmune hepatitis, hereditary hemochromatosis, Wilson’s disease, alpha-1-antitrypsin deficiency and present or previous active infection with HBV and HCV were ruled out using standard clinical and laboratory evaluation, as well as liver biopsy features.

Advanced fibrosis was defined in the presence of fibrosis stage F3-F4^40^, when liver biopsy was available. In HCC patients with radiological diagnosis, advanced fibrosis was defined in the presence of clinical, endoscopic or ultrasonographic signs of portal hypertension or cirrhosis (n = 46), or of liver stiffness ≥8.4 kPa evaluated by elastometry (n = 3), or by a positive NAFLD fibrosis score (n = 5)^41^. Obesity was defined when BMI > 30 Kg/m^2^. All NAFLD patients without HCC included in the study underwent liver biopsy, while among those who developed HCC, fibrosis staging was histologically performed in 78 (59%) of the cases. Clinical features of subjects included according to the presence of HCC are shown in Table 1.

The UK NAFLD cohort comprising HCC cases and controls recruited at a single tertiary centre (the Newcastle upon Tyne Hospitals NHS Foundation Trust, UK) has previously been described^21^. The study had all the necessary ethical approvals and all participants gave informed consent. For this study, we considered patients for whom liver disease staging scored according to the NASH CRN histopathological system and DNA samples for *MBOAT7* genotyping were available. Clinical features of the 358 individuals analyzed in this study are presented in Table S5.

We next evaluated the impact of the rs641738 variant on HCC risk in an Italian multicenter cohort of non-cirrhotic patients with chronic hepatitis C (CHC; n = 597) and alcoholic liver disease (ALD; n = 524). CHC patients were from the well-described histological Milan CHC cohort^42, 43^, whereas HCC cases were previously described by our group (Milan HCC cohort, where presence of cirrhosis was carefully assessed)^44^.

ALD patients with and without HCC included patients from the Milan center, which were partly described previously (again the Milan HCC cohort), and for whom liver disease was evaluated by histology or as described above for NAFLD^44^. We also considered consecutive individuals, who were admitted to the Outpatient Clinic at the Department of Clinical Medicine, Policlinico Umberto I, Rome for alcohol abuse or dependence between 2005 and 2014, for whom DNA samples were still available and genotyping was successful^45^. At-risk alcohol consumption was defined as ≥3/2 alcohol units per day for M/F, respectively. Cirrhosis was ruled out based on the presence of at least one of the following features: (i) current or past cirrhosis complications; (ii) the presence of at least two parameters among hyperbilirubinaemia, hypoalbuminaemia, prolonged prothrombin time, low platelet count, irregular liver surface at ultrasound/CT, reduced portal vein flow at ultrasound, gastroesophageal varices at endoscopy, or by histological analysis. Individuals with other coexistent liver diseases were excluded. Clinical features of this cohort are presented in Table S6.

The study protocol was conformed to the ethical guidelines of the 1975 Declaration of Helsinki, was approved by the Ethical Committee of the Fondazione IRCCS Ca’ Granda of Milan, as well as by the other involved Institutions, and was performed according to the recommendations of the hospitals involved. Informed consent was obtained from each patient.

### Genotyping

Patients were genotyped for rs738409 (*PNPLA3* I148M) rs58542926 (*TM6SF2* E167K), as previously described^27^. The rs641738 (located within *TMC4* coding sequence, chr19:54676763 positive strand, p.G17E protein variant) and rs8736 (located within the 3′-untranslated region – UTR – of *MBOAT7*) *MBOAT7/TMC4* locus genotyping has been performed in duplicate by TaqMan 5′-nuclease assays at the Metabolic Liver Disease lab, at the University of Milan (Life Technologies, Carlsbad, CA). Genotyping success rate was >98%. The duplicate genotype concordance rate was 100%. Genotyping was confirmed by direct sequencing of random samples, and concordance rate was 100% (primers are available upon demand).

### Gene expression analysis

Expression of MBOAT7 and TMC4 was determined in two different subsets of patients. The first one was made of 98 severely obese patients, with a very low prevalence of advanced liver fibrosis, and has previously been described in details^31^. This was used to analyze the association of a 3′-untranslated region *MBOAT7* locus variant, rs8736 possibly influencing MBOAT7 mRNA stability, with MBOAT7 expression. The second one was made up of 47 patients from the Hepatology service, and was characterized by a higher prevalence of liver fibrosis. Clinical features of these patients are presented in Table S4. This was used to evaluate the impact of liver fibrosis on the association between the rs641738 variant and MBOAT7 expression.

MBOAT7 expression was quantified as previously described^31^. Association analysis between rs641738 variant (additive model) and gene expression, and linkage with rs8736 were conducted by the PLINK v1.07 genetic analysis software.

### Statistical analysis

For descriptive statistics, continuous variables are shown as mean and standard deviation or median. Categorical variables are presented as number and proportion. The OR of HCC or severe fibrosis per *MBOAT7* rs641738 T alleles and other risk factors were estimated by logistic regression models, assuming an additive effect of genetic variants, and adjusted for clinical risk factors (the major known clinical risk factors for NAFLD-HCC in previous studies) as specified, *PNPLA3* I148M and *TM6SF2* E167K genotypes (clinical and genetic factors previously associated with or candidate for liver disease evolution to HCC)^21, 26^. A NAFLD-HCC risk score was developed according to a previously described procedure^41^.

Statistical analyses were carried out using the JMP 13.0 Statistical analysis software (SAS Institute, Cary, NC), R statistical analysis software version 3.3.2 (http://www.R-project.org/), and PLINK v1.07^46^. P-values < 0.05 were considered statistically significant. The study methods and results have been reported according to the STROBE/STREGA guidelines for genetic association studies.

## Electronic supplementary material

Supplementary material
