# Supplementary material for: MBOAT7 rs641738 variant and hepatocellular carcinoma in non-cirrhotic individuals
Source: Sci Rep. 2017 Jul 3;7:4492. doi: 10.1038/s41598-017-04991-0 (PMC5495751; doi:10.1038/s41598-017-04991-0)

MBOAT7 rs641738 variant and hepatocellular carcinoma in non-cirrhotic individuals

Benedetta Donati, Paola Dongiovanni, Stefano Romeo, Marica Meroni, Misti McCain, Luca Miele, Salvatore Petta, Silvia Maier, Chiara Rosso, Laura De Luca, Ester Vanni, Stefania Grimaudo, Renato Romagnoli, Fabio Colli, Flaminia Ferri, Rosellina Margherita Mancina, Paula Iruzubieta, Antonio Craxi, Anna Ludovica Fracanzani, Antonio Grieco, Stefano Ginanni Corradini, Alessio Aghemo, Massimo Colombo, Giorgio Soardo, Elisabetta Bugianesi, Helen Reeves, Quentin M. Anstee, Silvia Fargion, Luca Valenti.

Supplementary material

Supplementary tables

Table S1. Clinical and genetic features of 132 Italian patients with NAFLD-HCC stratified by the presence of severe hepatic fibrosis at diagnosis.

|  | Severe fibrosis (F3-F4 vs. F0-F2) | | p |
| --- | --- | --- | --- |
|  | Yes (n=111, 83%) | No (n=21, 17%) |  |
| Age, years | 66.3±8.3 | 68.3±12.3 | 0.49 |
| Sex, Female | 24 (22) | 1 (5) | 0.040 |
| Obesity, yes | 31 (28) | 8 (38) | 0.43 |
| T2DM, yes | 70 (63) | 14 (67) | 0.97 |
| *PNPLA3*, I148M |  |  | 0.052 |
| I/I | 22 (20) | 9 (43) |  |
| I/M | 49 (44) | 6 (29) |  |
| M/M | 40 (36) | 6 (27) |  |
| *TM6SF2*, E167K |  |  | 0.27 |
| E/E | 94(84) | 15 (71) |  |
| E/K | 14 (14) | 5 (24) |  |
| K/K | 3 (3) | 1 (5) |  |
| *MBOAT7*, rs641738 C>T |  |  | 0.006 |
| C/C | 25 (23) | 1 (5) |  |
| C/T | 60 (53) | 9 (43) |  |
| T/T | 26 (24) | 11 (52) |  |

(): % values; T2DM: type 2 diabetes mellitus. Comparisons were performed by logistic regression setting severe fibrosis as dependent variable, and the association with the genetic variant were analyzed assuming additive models.

Table S2. Clinical features in 765 Italian patients with NAFLD stratified by *MBOAT7* rs641738 polymorphism according to HCC diagnosis.

|  | Hepatocellular carcinoma | | | | | | | | Overall | | | |
| --- | --- | --- | --- | --- | --- | --- | --- | --- | --- | --- | --- | --- |
|  | Yes | | | | No | | | |  | | |  |
|  | rs641738 | | | p | rs641738 | | | p | rs641738 | | | p |
|  | C/C | C/T | T/T |  | C/C | C/T | T/T |  | C/C | C/T | T/T |  |
| n= | 26 | 69 | 37 |  | 213 | 285 | 135 |  | 239 | 354 | 172 |  |
| Age, years | 68±10 | 66±9 | 68±8 | 0.36 | 47±12 | 48±12 | 48±14 | 0.84 | 49±14 | 51±13 | 52±15 | 0.10 |
| Sex, Female | 5 (19) | 14 (20) | 6 (16) | 0.72 | 52 (24) | 68 (24) | 43 (32) | 0.17 | 57 (24) | 82 (23) | 49 (28) | 0.33 |
| Obesity, yes | 4 (15) | 24 (35) | 12 (33) | 0.035 | 66 (31) | 94 (33) | 42 (31) | 0.92 | 70 (29) | 117 (33) | 54 (31) | 0.36 |
| T2DM, yes | 15 (58) | 43 (62) | 26 (70) | 0.29 | 40 (19) | 58 (20) | 26 (19) | 0.92 | 55 (23) | 101 (28) | 52 (30) | 0.050 |
| Severe fibrosis, F3-F4 | 25 (96) | 59 (85) | 27 (73) | 0.006 | 30 (14) | 41 (14) | 27 (20) | 0.17 | 55 (23) | 100 (28) | 54 (31) | 0.052 |

( ): % values; T2DM: type 2 diabetes mellitus. Comparisons were performed by logistic regression setting HCC as dependent variable, and the association with the *MBOAT7* variant was analyzed assuming an additive model.

Table S3. Frequency distribution of the rs641738 C>T variant in 243 healthy individuals with available DNA samples, normal liver enzymes with low probability of steatosis*. HWE p=0.36.

| rs61738 genotype | N= | % |
| --- | --- | --- |
| C/C | 78 | 32 |
| C/T | 113 | 45 |
| T/T | 52 | 23 |

* The full cohort was described in: Rametta R, Ruscica M, Dongiovanni P, Macchi C, Fracanzani AL, Steffani L, Fargion S, Magni P, Valenti L. Hepatic steatosis and PNPLA3 I148M variant are associated with serum Fetuin-A independently of insulin resistance. Eur J Clin Invest. 2014 Jul;44(7):627-33. doi: 10.1111/eci.12280.

Table S4. Clinical and genetic features of 47 patients from the Milan Hepatology service, for whom hepatic gene expression analysis was available.

|  | Mean±SD; n (%) |
| --- | --- |
| Age, years | 48±10 |
| Sex, Female | 13 (28) |
| BMI, Kg/m^2^ | 30.3±5.1 |
| T2DM or IFG | 18 (38) |
| NASH, yes | 39 (83) |
| Fibrosis, stage F2-F4 | 26 (55) |
| *PNPLA3*, I148M | 11/23/13  (23/50/28) |
| *TM6SF2*, E167K | 35/12  (74/26) |
| *MBOAT7/TMC4*, rs641738 C>T | 22/19/6  (47/40/13) |

( ): % values; BMI: body mass index; T2DM: type 2 diabetes mellitus; NASH: nonalcoholic steatohepatitis.

Table S5. Clinical features of 358 non-cirrhotic patients from the UK NAFLD validation cohort.

| HCC | Yes | No |
| --- | --- | --- |
| n= | 20 | 338 |
| Age, years | 52±13 | 53.2±12.0 |
| Sex, Female | 2 (9) | 141 (42) |
| BMI, Kg/m^2^ | 29.1±5.5 | 34.2±7.1 |
| IFG/T2D, yes | 20 (98) | 177 (52) |
| *MBOAT7/TMC4*, rs641738 C>T | 4/11/5  (20/55/25) | 97/179/62  (29/53/18) |

(): % values; HCC: hepatocellular carcinoma, BMI: body mass index, IFG/T2D: impaired fasting glycemia or type 2 diabetes.

Table S6. Clinical and genetic features of 1121 patients with chronic hepatitis C (CHC) and alcoholic liver disease (ALD) without cirrhosis.

|  | Overall | CHC | ALD |
| --- | --- | --- | --- |
| n= | 1121 | 597 | 524 |
| Age, years | 52±13 | 60±13 | 47±12 |
| Sex, Female | 387 (34) | 258 (43) | 129 (25) |
| HCC, yes | 25 (2) | 13 (2) | 12 (2) |
| *PNPLA3*, I148M | 566/447/108  (50/40/10) | 318/224/55  (53/38/9) | 248/223/53  (47/43/10) |
| *TM6SF2*, E167K | 993/127/1  (82/12/0) | 547/50/0  (92/8/0) | 446/77/1  (85/15/0) |
| *MBOAT7/TMC4*, rs641738 C>T | 329/525/267  (29/47/24) | 178/266/153  (30/44/26) | 151/259/114  (29/49/22) |

(): % values; HCC: hepatocellular carcinoma.

Table S7. Independent predictors of hepatocellular carcinoma (HCC) in 597 with chronic hepatitis C (CHC) and 524 with alcoholic liver disease (ALD) stratified by the presence of cirrhosis.

| Chronic hepatitis C | | | | |
| --- | --- | --- | --- | --- |
|  | HCC | |  |  |
|  | No (n=584) | Yes (n=13) | p value | p value* |
| *PNPLA3* I148M | 313/219/52  (54/37/9) | 5/5/3  (38/39/24) | 0.18 | 0.22 |
| *TM6SF2* E167K | 538/46  (92/8) | 9/4  (69/31) | 0.032 | 0.008 |
| *MBOAT7*  rs641738 C>T | 177/259/148  (30/45/25) | 1/7/5  (8/54/38) | 0.087 | 0.071 |
| Alcoholic liver disease | | | | |
|  | HCC | |  |  |
|  | No (n=512) | Yes (n=12) | p value | p value* |
| *PNPLA3* I148M | 245/218/49  (48/43/9) | 3/5/4  (25/42/33) | 0.054 | 0.041 |
| *TM6SF2* E167K | 435/76/1  (85/15/0) | 11/1  (92/8) | 0.58 | 0.41 |
| *MBOAT7* rs641738 C>T | 150/251/111  (29/49/22) | 1/8/3  (8/67/25) | 0.12 | 0.090 |

(): % values; * Comparisons were performed by logistic regression setting HCC as dependent variable, and associations with genetic risk variants were analyzed assuming additive models. Adjusted for age, sex, and *PNPLA3*, *TM6SF2* and *MBOAT7* genetic variants.

Supplementary figures

Figure S1. Receiving operating characteristic (ROC) curve for the combined risk score for predicting hepatocellular carcinoma in NAFLD patients. A combined risk score considering acquired and genetic risk factors was developed to predict HCC: 1 / (1 + e ^ - ((-12.588 + (0.162 * age) + (0.404 * Sex: 1 if male, -1 if female) + (0.259 * Obesity: 1 present, -1 absent) + (0.587 * T2DM: 1 present, -1 absent) + (1.299 * Severe Fibrosis: 1 yes, -1 no) + (0.442 * number of risk alleles))). The model had a 0.96 area under the ROC curve. The optimal cutoff (identified by the green straight line tangent to the ROC curve) had 96% sensitivity and 89% specificity for HCC in the present cohort.


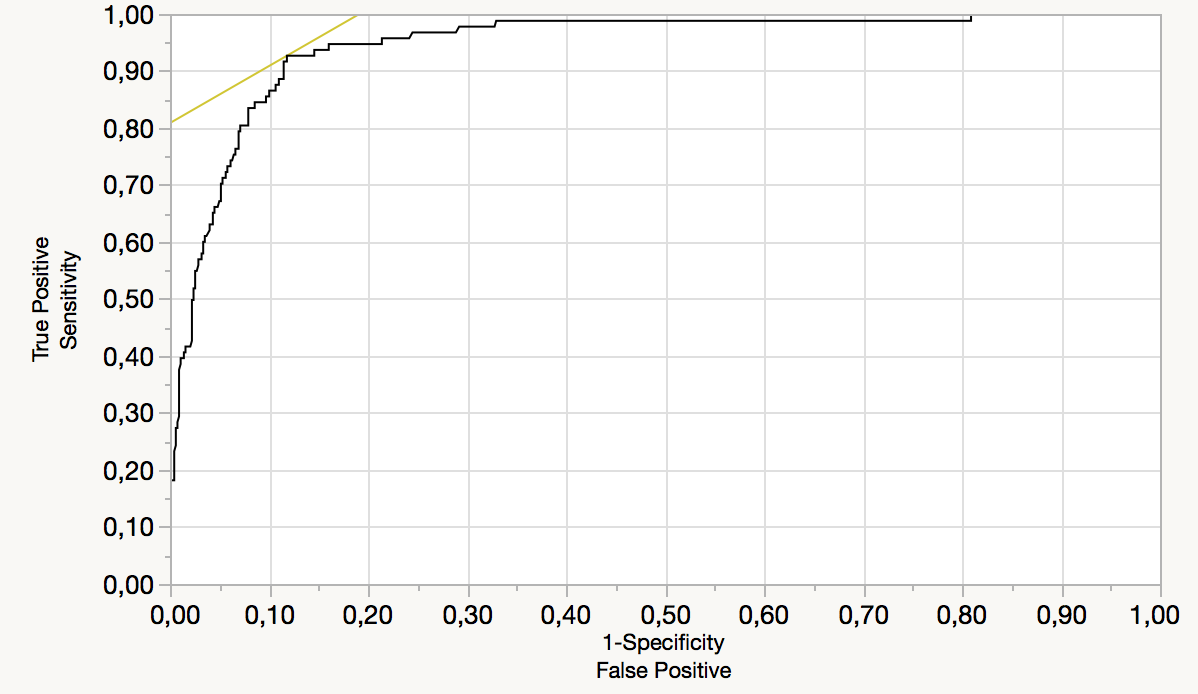

Supplement: Supplementary file 1 — Supplementary material [file 41598_2017_4991_MOESM1_ESM.doc]
